# Serial processing of proximity groups and similarity groups

**DOI:** 10.3758/s13414-024-02861-2

**Published:** 2024-03-11

**Authors:** Robert C. G. Johansson, Rolf Ulrich

**Affiliations:** Fachbereich Psychologie, Eberhard Karls Universität Tübingen, Schleichstraße 4, 72076 Tübingen, Germany

**Keywords:** Perceptual grouping, Similarity, Proximity, Gestalt, Serial and parallel processing

## Abstract

Proximity and feature similarity are two important determinants of perceptual grouping in vision. When viewing visual scenes conveying both grouping options simultaneously, people most usually detect proximity groups faster than similarity groups. This article demonstrates that perceptual judgments of grouping orientation guided by either proximity or contrast similarity are indicative of a sequential organization of grouping operations in the visual pathway, which lends a temporal processing advantage to proximity grouping (Experiment 1). Invoking the double-factorial paradigm, latent cognitive architecture for perceptual grouping is also investigated in a task with redundant signals (Experiment 2). Reaction time data from this task is assessed in terms of the race model inequality, workload capacity analysis, and interaction contrasts of means and survivor functions. Again, empirical benchmarks indicate serial processing of proximity groups and similarity groups, with a self-terminating stopping rule for processing. A subset of participants exhibit atypical performance metrics, hinting at possible individual differences in configural visual processing.

The human visual system is highly fluent in parsing optical input into structured and meaningful units, commonly termed perceptual groups (Wertheimer, 1923; Wagemans et al., 2012; Kubovy et al., 1998) . The Gestalt laws governing perceptual organization in vision include grouping by closeness in space (law of proximity) and grouping by shared featural properties such as matching contrast level or hue (law of similarity). An important theoretical issue in vision science is whether a common set of mental operations supports all types of grouping or whether distinct operations govern particular Gestalt laws (Houtkamp & Roelfsema, 2010; Roelfsema & Houtkamp, 2011; Zucker et al., 1983; Palmer et al., 2003). Vision researchers have therefore tried to characterize the cognitive mechanisms of perceptual grouping in terms of their structural locus (Grassi et al., 2016; Grossberg & Williamson, 2001; Han et al., 2002, 2001; Fang et al., 2008; Huberle & Karnath, 2012) as well as the time-course of different grouping operations relative to one another (Baylis & Driver, 1992; Pomerantz & Garner, 1973; Pomerantz & Schwaitzberg, 1975; Palmer et al., 2003; Han & Humphreys, 1999; Han, 2004; Schmidt & Schmidt, 2013; Wannig et al., 2011; Rashal et al., 2017; Razpurker-Apfeld & Kimchi, 2007).

A prominent finding from this research is that proximity grouping has a relatively fast time-course when compared to similarity grouping through shared attributes such as similar shape, similar color, and so forth (Ben-Av & Sagi, 1995; Luna et al., 2016; Villalba-García et al., 2018; Trick & Enns, 1977). We shall refer to this empirical finding as the *proximity advantage*. This article is concerned with elucidating the factors at play in the chain of visual processing events which give rise to the proximity advantage. To this end, we investigated people’s ability to judge the orientation of perceptual groups defined by proximity and contrast similarity. This ability was examined under conditions of focused attention (Experiment 1) and divided attention (Experiment 2). Of particular theoretical interest is whether proximity groups and contrast similarity groups are processed in serial or in parallel, and the extent to which these two grouping mechanisms operate independently.

## Proximity advantage

**Fig. 1:**
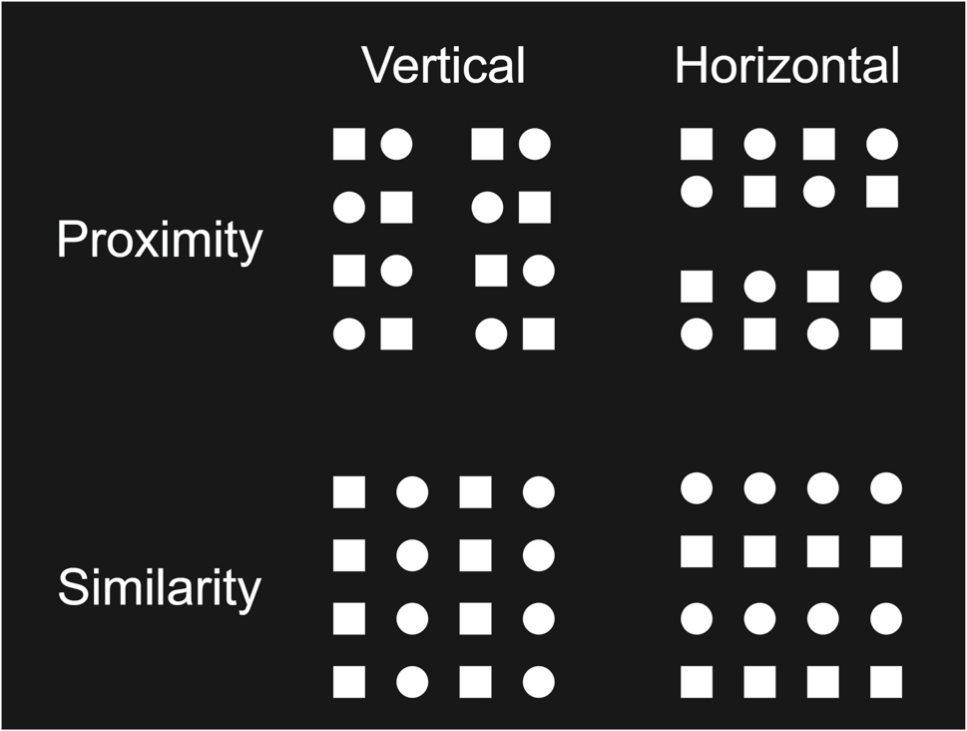
Schematic illustration of stimulus materials from Han et al. (2001). The *upper row* depicts arrays with proximity grouping, and the *lower row* depicts arrays with similarity grouping. Whenever one of the leftmost arrays are presented, participants had to respond “vertical”. When one of the rightmost arrays are presented, they had to respond “horizontal”

A key source of evidence for the proximity advantage comes from a series of studies conducted by Han and colleagues (Han & Humphreys, 1999; Han et al., 2001, 2002; Han, 2004). In one experiment (Han et al., 2001, Experiment 1), the authors investigated the time-course of perceptual grouping by proximity and similar shape. Participants were presented with visual arrays containing simple geometrical forms (circles and squares) organized in a periodic pattern. We show these arrays in Fig. 1 for the reader’s convenience. On each trial, the local elements of the array were organized into either a horizontal or a vertical configuration such that the perceptual groups formed either rows or columns. This was achieved by periodically spacing the elements further from some neighboring elements and closer to others (proximity grouping), or by organizing the pattern into repeated stripes of circles and squares (similarity grouping). For each array, people were required to indicate if the grouping orientation was vertical or horizontal with a speeded button press. Event-related potentials (ERPs) associated with the two types of grouping were measured together with response times (RTs) and error rates. Their results can be recapitulated as follows:

Speeded orientation judgments of proximity grouping were on average faster and more accurate than similarity grouping judgments. Proximity grouping also elicited an early positivity difference over the medial occipital cortex. This early positivity was not present for arrays grouped by shape similarity. Instead, similarity grouping was associated with a longer latency ERP component over the lateral occipital cortex. Roughly comparable results were obtained in another study investigating the grouping cues of proximity and color similarity (Han et al., 2002) although this study did not observe a difference in accuracy for proximity and similarity grouping. In broad strokes, these findings are consistent with the notion of an earlier structural locus of visual processing for proximity groups relative to similarity groups, which manifests both behaviorally and electrophysiologically as a proximity advantage in the temporal domain.

It has been suggested that the faster time-course of proximity grouping relative to similarity grouping reflects constraints on visual processing imposed by the hierarchical arrangement of subdivisions in the human visual pathway (Sasaki, 2007; Huang, 2015; Han et al., 2005; Quinlan & Wilton, 1998). Under this view, grouping cues such as proximity are seen to be fairly low-level in the sense that they are detected already at the earliest stage of perceptual organization. This processing stage is assumed to be primarily concerned with spatial configuration, but to be strikingly insensitive to cues for similarity grouping, such as whether a given region of space is red or blue, for example. Only at a subsequent processing stage are proximity groups complemented by perceptual analysis of contingencies in visual dimensions such as similarity of color or shape. This state of affairs then lends a temporal processing advantage to proximity grouping over similarity grouping.

The above sketched account of perceptual grouping operations can then be conceived as a serial processing chain: Proximity groups are detected first, and similarity groups later. This sequential conception of perceptual grouping operations in the visual pathway might admittedly oversimplify matters because it posits that the flow of information is strictly feed-forward. Empirically, top-down control and recurrent feedback are believed to influence perceptual grouping (Roelfsema, 2006; Kon & Francis, 2022; Dobbins & Grossmann, 2002; Beck & Palmer, 2002; Fang et al., 2008). However, the extent to which feed-backward interactions support perceptual grouping under conditions of time pressure (such as in standard RT tasks when people are instructed to respond very fast) is an open question. One possible way to investigate whether proximity groups and similarity groups are processed in serial is to examine people’s ability to process more complex visual stimuli, which sometimes contain conflicting configural information, as explained below.

### Congruence effect

**Fig. 2:**
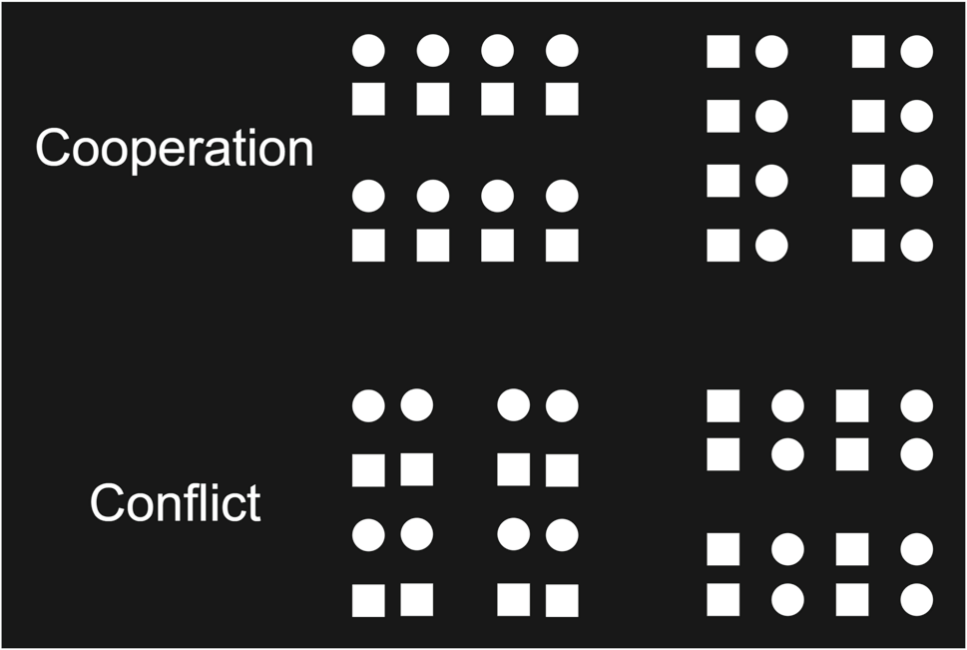
Schematic illustration of stimulus materials from Han (2004). In the *upper row* of arrays, grouping by proximity and grouping by shape similarity indicate the same global stimulus configuration in cooperative fashion. In the *lower row* of arrays, proximity and shape similarity indicate conflicting stimulus configurations

A paradigm for studying interactions between grouping cues was developed by Han (2004). People were shown visual arrays wherein the individual elements were grouped together on the basis of proximity and similarity, such that each grouping cue served to induce either a vertical or a horizontal grouping orientation. Participants had to attend to the orientation of a target grouping cue (while ignoring the orientation of the non-target cue as best they could) and indicate whether the target group was arranged horizontally or vertically. Sometimes the two cues indicated the same orientation (cooperation trials) and sometimes opposite orientations (conflict trials; see upper and lower panels of Fig. 2, respectively). Again, proximity orientation judgments were particularly fluent in terms of both speed and accuracy (the proximity advantage). More pertinent, however, responses were also slower and more error prone when the two grouping orientations conflicted as compared to when they were in cooperation. This congruence effect was more pronounced when similarity was the target group than when proximity was the target group.

The attenuated congruence effect for proximity group orientation judgments is further consistent with the idea that proximity and similarity groups are processed in serial. To intuit why, consider the following analogy of two factory workers in the act of assembling light bulbs next to a conveyor belt: First, worker number one determines whether the caps are properly fitted to the bulb (processing stage 1). The second worker then evaluates each light bulb individually by screwing it into an electrical socket (processing stage 2). If the first worker sends a bulb with an improperly fitted cap (a conflicting array) down the conveyor belt, then the second worker might encounter some difficulty fitting the bulb into the socket (slower processing). However, in the contrasting case, where the second worker makes an error while screwing a properly fitted light bulb into the socket, this does not entail a problem for the first worker because the conveyor belt only rolls forward and never in reverse (the manufacturing process is sequential feed-forward). Assume that a similar serial processing architecture holds for perceptual grouping, so that proximity groups are processed prior to similarity groups: We would then expect that the conflicting proximity groups can interfere with judgments of similarity group orientation, but the conflicting similarity groups should conversely not interfere with judgments of proximity group orientation, similar to what was observed by Han (2004).

A caveat that complicates the interpretation of these data is that Han (2004) did not use a neutral condition. By neutral condition, we mean a set of trials in which only a single grouping cue is present in the array. Comparing performance metrics on conflict and cooperation trials relative to a neutral baseline might provide new clues about the origins of the congruence effect which constrain theoretical accounts of perceptual grouping by proximity and similarity. Three different interpretations of the congruence effect seem possible: First, two cooperating cues might facilitate performance by conveying convergent information about orientation to the observer (a facilitation effect). Second, two conflicting cues might interfere with performance by providing discordant information (an interference effect); and finally, some combination of facilitation and interference could also mimic the observed pattern of results. These three accounts cannot currently be distinguished from the data at hand.

To summarize, it seems worthwhile to further investigate whether the congruence effect is the same when proximity is the task-relevant grouping cue and when similarity is task-relevant. The study by Han (2004) found that the congruence effect was attenuated for proximity judgments, implying that possible cross-talk between processing stages might not be strictly symmetrical. This asymmetry is seemingly in line with the serial processing account of the proximity advantage, according to which the faster time-course of proximity grouping reflects the precedence of spatial analysis in early visual processing. It is also of interest to determine if the congruence effect is due to interference or facilitation (or both) from the task-irrelevant grouping dimension. Experiment 1 aimed to test whether cross-talk between proximity grouping and similarity grouping is bidirectional, and whether said cross-talk interferes with or facilitates performance.

**Fig. 3:**
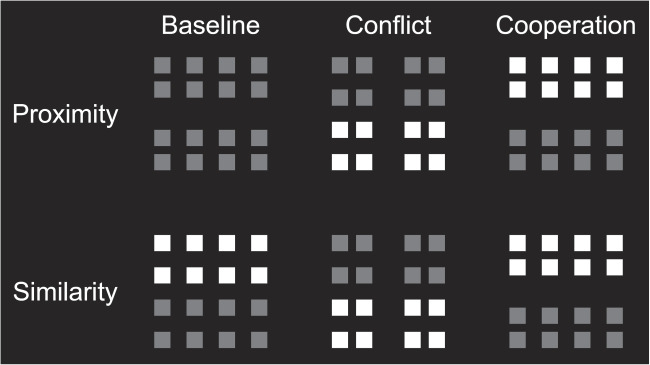
Schematic depiction of stimulus materials from Experiment 1. The *upper* row depicts representative array configurations for proximity judgments, and the *lower row* depicts the corresponding arrays for similarity judgments. From left to right, the array types depict baseline, cooperation and conflict trials, respectively. Please note that with the exception of baseline trials, the array configurations were the same for proximity judgments and similarity judgments

## Experiment 1 (Focused attention)

This preregistered experiment (https://osf.io/tjrw5) relied on a grouping orientation judgment task similar to Han (2004). The purpose was to study interactions between grouping by proximity and grouping by contrast similarity. Participants were presented with visual arrays as depicted in Fig. 3 and were instructed to judge the orientation of the target group as either vertical or horizontal. A novel tweak to the experimental design was the inclusion of a baseline condition (leftmost panels) in which only a single grouping cue was present in the array. The baseline condition was intended to serve as an empirical benchmark against which to compare performance on conflict and cooperation trials (analogous to the ‘target alone’ condition in Eriksen and Eriksen, 1974). This should enable us to distinguish between the three possible accounts of the congruence effect, namely facilitation, interference, or both. We were also interested in whether congruence effects are the same for proximity and similarity judgments.

### Method

#### Participants

Sixty individuals were recruited from a native German-speaking participant pool via the online service Prolific (www.prolific.com). Six of them were excluded due to exhibiting low (< 90 %) overall accuracy of responding, yielding a final sample size of 54 individuals (39 males) with a mean age of 30 years (age range, 18–64 years). All participants were naïve to the purpose of the experiment and reported normal or corrected-to-normal vision. They completed a single session each at a time and place of their choosing, with the only constraint being that they follow through the procedure on a personal computer rather than on a phone or a tablet.

The target sample size was determined through a priori power analysis based on iterative Monte Carlo simulation. The power analysis aimed to determine the sample size sufficient to achieve what corresponds to 95 % frequentist power for all pertinent research hypotheses. Data were simulated and fitted 10,000 times to confirmatory Bayesian statistical models in steps of increasing sample size with the Bayes factor (*BF*) criterion threshold set to substantial evidence ($BF > 3$). The simulation was informed by extensive pilot testing of the experiment.

#### Apparatus and stimuli

Stimulus presentation and response recording was controlled by a script in PsychoPy software (Peirce et al., 2019) and implemented online on the launch platform Pavlovia (https://pavlovia.org/). Participants responded by making one out of two possible button presses on their keyboards; the ‘up’ arrow to indicate a vertical grouping arrangement (columns) or the ‘right’ arrow to indicate a horizontal grouping arrangement (rows). Visual stimuli were quadratic arrays containing 16 gray monochrome squares organized in a 4 $\times$ 4 grid. The arrays were presented centrally on the computer monitor against a dark background. The global configuration of the array was manipulated by adjusting the proximity of the squares, or their relative contrast level, or both. The purpose of these manipulations was to induce perceptual grouping by dividing the arrays into either an upper and a lower subset (rows) or a left and right subset (columns) such that the array was organized into groups along the horizontal or vertical main axes, respectively. Stimulus materials are depicted schematically in Fig. 3.

To ensure that the visual stimuli retain proportionality despite variability in monitor size and screen resolution, the stimulus metric was defined relative to screen height. Each square measured 0.04 height units in diameter. Neighboring squares were separated by 0.04 height units under conditions of no proximity grouping. By extrapolation, the entire array measured 0.28 height units in diameter. Proximal (grouped) squares were separated by 0.025 height units, and distal (ungrouped) squares were separated by 0.07 units. Dim squares were defined as middle gray in RGB color space [0,0,0] and bright squares were defined as white [1,1,1]. The squares were presented against a black [-1,-1,-1] background following the offset of a mid-gray crosshair. A random stimulus latency component was sampled from a zero-truncated Gaussian with a mean and standard deviation of 1000 ms to avoid rhythmic responses. This random latency was added to a constant foreperiod of 500 ms.

#### Procedure and design

Participants were tested in a setting of their choice in a single session comprising 288 trials. They were greeted with written instructions on the monitor underscoring the importance of responding as fast and as accurately as possible. Trials were separated into four experimental blocks comprising 72 trials each, preceded by two practice blocks comprising 24 trials each. In two experimental blocks, the task-relevant dimension was the contrast level of the squares (similarity grouping), and in the other two blocks the target dimension was the spatial separation between squares (proximity grouping). Contrast similarity and proximity were manipulated factorially, either in isolation on baseline trials or in combination on cooperation and conflict trials. This yielded six unique stimulus conditions: (a) similarity baseline, (b) similarity cooperation, (c) similarity conflict, (d) proximity baseline, (e) proximity cooperation, and (f) proximity conflict. Participants were instructed to attend to one grouping dimension at a time in alternating blocks. Conflict level was intermixed within blocks. Stimuli were presented in pseudo-randomly constructed cycles of 12 trials each, counterbalanced for the orientation of the target dimension (vertical or horizontal) and the manner in which similarity grouping divided the array into regions of high and low contrast. In total, 48 experimental trials per condition (12 trials in each of the six stimulus conditions times four blocks) were presented. At the end of each practice and experimental block, the procedure was paused, and task instructions were re-presented. An opportunity to rest was provided between blocks. In all respects, the experiment was conducted in accordance with the guidelines for human subject research laid down by the Tübingen Ethics Committee for Psychological Research and the Declaration of Helsinki.

#### Data analysis

First, the RT data were inspected for outliers. All responses faster than 200 ms were considered anticipatory, and responses slower than 1500 ms were considered misses. Anticipations and misses were removed from further analysis. Few responses were found to be too fast (0.1 %) or too slow (2.34 %). Mean error rates and mean-corrected RTs for each participant were then entered separately into two 2 $\times$ 3 (task-relevant dimension $\times$ conflict level) repeated measures Bayesian analyses of variance (BANOVAs) with participant ID as a random factor. The two levels of the factor *grouping dimension* were proximity and contrast similarity, and the three levels of the *conflict level* factor were baseline, cooperation, and conflict. Bayes factors (*BF*s) were computed with the *BayesFactor* package (Morey & Rouder, 2022) by comparing the evidence for BANOVA models incorporating main and interaction effects to intercept-only models. The predetermined criterion threshold for evidential strength was $BF > 3$, typically taken to indicate moderate evidence for a hypothesis (Lee & Wagenmakers, 2013).

Graphical reliability measures for the error rate and RT data are reported in terms of their within-subjects $95~\%$ highest density intervals (95 $\%$ HDIs). These HDIs were calculated in two steps; first by centering the data for each participant and condition as advised by Cousineau (2005) and Loftus and Masson (1994). Then, the mean-centered data were entered into Bayesian linear models to compute parameter estimates using *RStan* software (Stan Development Team, 2023).

All Bayesian analyses conducted using simulation are based on 1000 warm-up steps, and 10,000 sampled steps, for each of three independent chains. These chains showed little autocorrelation, as determined with the $\hat{R}$ convergence statistic (Gelman & Rubin, 1992), which was found to be less than 1.01 in every case. The reported parameter estimates should therefore be highly representative of their posterior distributions. All analyses were conducted in the R software for statistical computing (R Core Team, 2022). The analysis code is available together with experimental data via the Open Science Foundation (https://osf.io/wt2gb/).

**Fig. 4:**
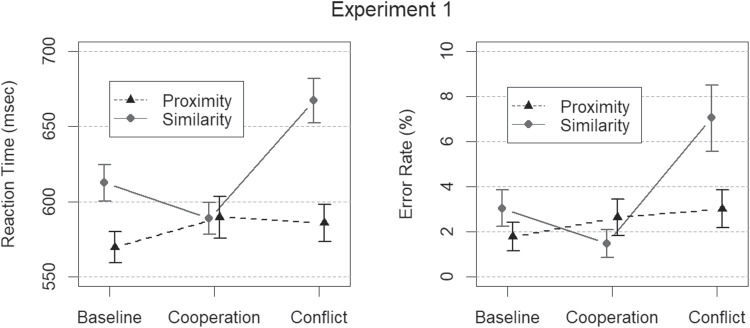
Results from Experiment 1 in terms of mean RTs (*left panel*) and mean error rates (*right panel*), depicted here as a function of grouping dimension (proximity or contrast similarity) and conflict level (baseline, cooperation and conflict). *Error bars* represent the 95 % HDIs of the posterior distributions of means

### Results and discussion

The error rate model incorporating main effects of conflict level ($BF > 10^{7}$) and grouping dimension ($BF = 27$) yielded extreme and strong evidence for a congruence effect and the proximity advantage, respectively. However, the best-fitting BANOVA model of error rates included a factorial interaction between grouping dimension and conflict level in addition to main effects ($BF>7 \times 10^{13}$). A similar pattern of results was apparent for RTs. Again, the evidence for main effects of grouping dimension ($BF > 4 \times 10^{7}$) and conflict level ($BF > 14,000$) on RTs suggested both a congruence effect and a proximity advantage. Yet, the best-fitting RT model incorporated an interaction between grouping dimension and conflict level as well as main effects of grouping dimension and conflict level. The evidential strength was very large ($BF > 2 \times 10^{18}$). Factorial plots of mean error rates and mean correct RTs are given in Fig. 4. Taken together, both error rates and RTs were indicative of a proximity advantage and a congruence effect. Interaction terms suggest a differential pattern of cross-talk for the two grouping dimension. Post hoc contrasts using Bayesian *t* tests further confirmed these findings. Regarding similarity judgments, RTs were shorter and more accurate on cooperation trials, intermediate on baseline trials, and longer and less accurate on conflict trials (all $BFs > 3$). For proximity judgments, both RTs and error rates were equivalent between cooperation and conflict trials ($BFs < \frac{1}{3}$). However, baseline proximity RTs were shorter compared to both cooperation trials and conflict trials ($BFs > 100$), and were less accurate on conflict trials relative to baseline ($BF=3.49$). The comparison of error rates for proximity judgments on baseline and cooperation trials was inconclusive ($\frac{1}{3}< BF < 3$).

In summary, Experiment 1 replicated two important behavioral benchmarks of human performance in speeded orientation judgments of multidimensional Gestalt stimuli reported in earlier studies (e.g., Han et al., 2001; Han et al., 2002 and Han, 2004). First, there was an overall proximity advantage, as indexed by how orientation judgments of proximity groups were both faster and less error-prone than orientation judgments of contrast similarity groups. The observed proximity advantage can therefore not be attributed to more reckless responding when proximity is the target dimension (i.e., a speed-accuracy trade-off). Second, we found evidence of a congruence effect indicating cross-talk between processing stages. Visual inspection of Figure 4 suggests that both candidate sources (interference and facilitation) give rise to the congruence effect when similarity grouping is the target dimension. Finally, and perhaps most pertinent for the present research, there was evidence of an asymmetric congruence effect, because judgments of proximity orientation were unaffected by whether contrast similarity grouping was in conflict or in cooperation. In our opinion, these findings strengthen the case that the proximity advantage reflects the involvement of an earlier visual processing stage involved in the detection of proximity groups relative to similarity groups. The asymmetric congruence effect in particular lends strong support to the notion of a two-stage serial model of grouping by proximity and similarity.

Several theoretical accounts of perceptual grouping have argued for the existence of two distinct stages of processing; an initial spatial analysis stage followed by a subsequent feature analysis stage (Huang, 2015; Sasaki, 2007; Yu et al., 2019; Quinlan & Wilton, 1998). An idea advanced by Quinlan and Wilton (1998) is that the output of the spatial analysis stage is a set of local clusters (proximity groups) whose features (e.g., color and shape) have not yet been processed. In the second stage, the feature values of the elements contained within each cluster are detected and compared with each other for possible matches. Features are only checked between clusters in the case of a within-cluster mismatch. Therefore, the serial processing account of perceptual grouping implies a slower time-course for similarity grouping when proximity and similarity cues are in conflict as observed in Experiment 1. A serial processing architecture can also account for different congruence effects for the two grouping dimensions: Because the local clusters are defined prior to the feature analysis stage, proximity judgments should not be affected by whether features within said clusters match or mismatch.

To reiterate, we argue that the results of Experiment 1 support a two-stage serial model of grouping by proximity and contrast similarity. Yet, the task of distinguishing serial and parallel processing based on behavioral data is notoriously difficult (Townsend, 1971, 1990; Townsend & Nozawa, 1997; Colonius & Vorberg, 1994; Vorberg & Ulrich, 1987). Therefore, invoking the double-factorial paradigm (Townsend & Nozawa, 1995; Wenger & Townsend, 2000; Houpt et al., 2014; Altieri et al., 2017; Houpt & Townsend, 2010, 2012), Experiment 2 aimed to test the serial processing account of grouping by similarity and proximity from a novel theoretical perspective as explained below. The reader familiar with the double-factorial method and associated analysis procedures for distinguishing serial and parallel processing might only skim the next section briefly or skip it entirely, as it provides an introductory overview of these procedures for a broader readership.

### Double-factorial paradigm

The double-factorial paradigm is an experimental methodology developed to characterize multi-channel processing systems in terms of latent cognitive architecture (serial, parallel, or coactive), combination or stopping rule for processing (exhaustive or self-terminating), workload capacity (limited, unlimited, or super-capacity), and possible stochastic dependencies between processing pathways (dependent or independent) (Altieri et al., 2017).

This is achieved by instructing people to monitor two sensory channels for incoming targets and to respond to these targets as fast as possible. On any given trial in this divided attention task, a target signal can appear in either a single channel (singleton trials) or in both channels simultaneously (redundant trials). For RT tasks with two response options, the targets in the two channels are most usually mapped to the same manual response on those trials where they appear together. In this regard, the two stimulus components are redundant, because each component independently conveys all the information necessary for the observer to respond. The double-factorial method is therefore an extension of the redundant signals paradigm (Miller, 1982; Raab, 1962) in which people’s ability to detect either of singleton signals A or B is compared to their ability to detect a redundant signal combination AB. In the context of the present experiment, the two signal components A and B denote grouping by proximity and grouping by similar contrast level, and their redundant combination AB denotes multidimensional Gestalt stimulus arrays containing both grouping cues. Importantly, the stimulus arrays used in Experiment 1 were not redundant in this respect because the two grouping cues sometimes afforded conflicting information about orientation to the participants.

The double-factorial derives its name from two critical factorial manipulations of the stimulus: the *redundancy manipulation* and the *salience manipulation*. The aforementioned redundancy manipulation is a manipulation of perceptual workload that concerns the number of targets (singleton vs. redundant) available to the observer. It allows one to contrast the RTs from singleton trials and redundant trials, and test the data against the predictions of a “horse race model” embodying parallel independent processing of information sources A and B with a self-terminating stopping rule for processing. This model metaphorically resembles a horse race in the sense that the two signals A and B race along separate channels towards an OR-gated response center on redundant trials. The signals are assumed independent in the sense that a target on channel A does not influence processing speed on channel B and vice versa. The fastest signal to be detected “wins” the race and independently triggers a manual response, yielding a statistical facilitation of RTs of magnitude

1 $$\begin{aligned} RT_{AB} = \min (RT_A, RT_B) \end{aligned}$$

where $RT_{A}$ and $RT_{B}$ are the RTs on singleton trials, and $RT_{AB}$ is the theoretically inferred RT on redundant trials. The race model therefore makes the decisive prediction that no response on redundant trials can be faster than the fastest response (or slower than the slowest response) on singleton trial. The upper bound for statistical facilitation, as given by the race model inequality (Miller, 1982), states that

2 $$\begin{aligned} P(RT_{AB} \le t) \le P(RT_{A} \le t) + P(RT_{B} \le t) \end{aligned}$$

where the left-hand term denotes the cumulative density function (CDF) for $RT_{AB}$ and the right-hand terms denotes the CDFs for $RT_{A}$ and $RT_{B}$, respectively. Violations of the race model inequality have often been used to refute serial and parallel processing models in favor of coactive models, where information is pooled across both channels to elicit a response (Miller, 1982).

Processing capacity is similarly assessed by contrasting performance on redundant trials relative to singleton trials. Following Wenger and Townsend (2000), a capacity coefficient $C_{OR}(t)$ can be computed from the ratio of the integrated hazard function for RTs from redundant trials over the sum of the integrated hazard functions for RTs from singleton trials, such that

3 $$\begin{aligned} C_{OR}(t) = \frac{H_{AB} (t)}{H_A (t) + H_B (t)} \end{aligned}$$

where the numerator denotes the integrated hazard function for RTs from redundant trials, and the denominator denotes the sum of the integrated hazard functions from singleton trials. The unitless metric $C_{OR}(t)$ denotes the empirically established rate of information processing on redundant trials relative to singleton trials, and can be compared to the theoretical predictions of the horse race model. The horse race model predicts that $C_{OR}(t)$ should equal unity across all values of $t > 0$, a benchmark termed *unlimited capacity*. In contrast, *limited capacity* is formally defined as a capacity coefficient that is significantly less than 1, while *super capacity* is defined as a capacity coefficient that is significantly greater than 1. When a system exhibits limited processing capacity or super capacity, this is an indication that processing pathways under consideration are not consistent with self-terminating, independent parallel processing. Workload capacity can be formally evaluated with the Houpt–Townsend test (Houpt & Townsend, 2012; Houpt et al., 2014).

The second manipulated factor alluded to in the epithet “double-factorial” concerns the salience of the two stimulus components, that is to say, whether the two targets on channels A and B are easy or difficult to detect. This factor serves to aid the experimenter in determining whether the two salience manipulations have additive effects on RT or not. Essentially, the two salience manipulations should have additive effects on RTs if they affect independent processing stages, but have interactive effects on RT if the two processing stages are not independent. In this regard, the salience manipulation is an outgrowth of the additive factors method of Sternberg (1969). Let *M* denote the mean RT on redundant trials when the targets on channels A and B are either low salience (*L*) or high salience (*H*). Following Sternberg (1969), one can compute a mean interaction contrast (MIC) as follows

4 $$\begin{aligned} MIC = M_{LL} - M_{LH} - M_{HL} + M_{HH} \end{aligned}$$

If the effects of increased grouping salience are additive, then Eq. 4 should equal zero. Correspondingly, the two factors are sub-additive if the MIC is negative, and super-additive if the MIC is positive.

Following Houpt and Townsend (2010), the survivor distributions functions belonging to the RT distributions from the four redundant signal combinations can also be subjected to a survivor interaction contrast (*SIC*) as follows

5 $$\begin{aligned} SIC(t) = S_{LL}(t) - S_{LH}(t) - S_{HL}(t) + S_{HH}(t) \end{aligned}$$

where the subscripts again indicate the presence of a low salience (*L*) or a high salience (*H*) target on channels A and B respectively, and *S*(*t*) denotes the survivor function for RTs belonging to that trial type. The shape of the SIC curve for values of $t > 0$ is further indicative of certain types of processing architectures (serial, parallel, or coactive) and stopping rules (exhaustive or self-terminating). When all of the aforementioned analysis methods for RTs are combined, they constitute a particularly powerful set of tools for distinguishing between serial, parallel, and coactive model architectures (Houpt et al., 2014; Altieri et al., 2017). We therefore sought to use the race model inequality, capacity analysis, the SIC test, and the MIC test, to further assess latent cognitive architecture subserving perceptual grouping by proximity and contrast similarity. How to interpret said contrasts of CDFs, hazard functions, survivor functions, and mean RTs, will be further elaborated on in the General discussion section ensuing this experiment.

## Experiment 2 (divided attention)

Invoking the double-factorial paradigm and associated methods for analyzing RT distributions, the goal of Experiment 2 was to further characterize visual information processing in the classification of multidimensional Gestalt stimuli. More specifically, the research aims of Experiment 2 were to evaluate: (a) if proximity grouping and contrast similarity grouping occur in serial or in parallel, (b) if the stopping rule for processing is exhaustive or self-terminating, (c) if processing capacity for such visual stimuli is limited, and (d) if proximity groups and contrast similarity groups are processed independently. These research questions were evaluated on the basis of a set of non-parametric procedures developed to aid the analysis of RT data garnered in the double-factorial paradigm as detailed above. To this end, the design of Experiment 1 was modified by removing all arrays containing conflicting grouping cues. In addition, two levels of grouping salience were employed for each grouping dimension.

**Fig. 5:**
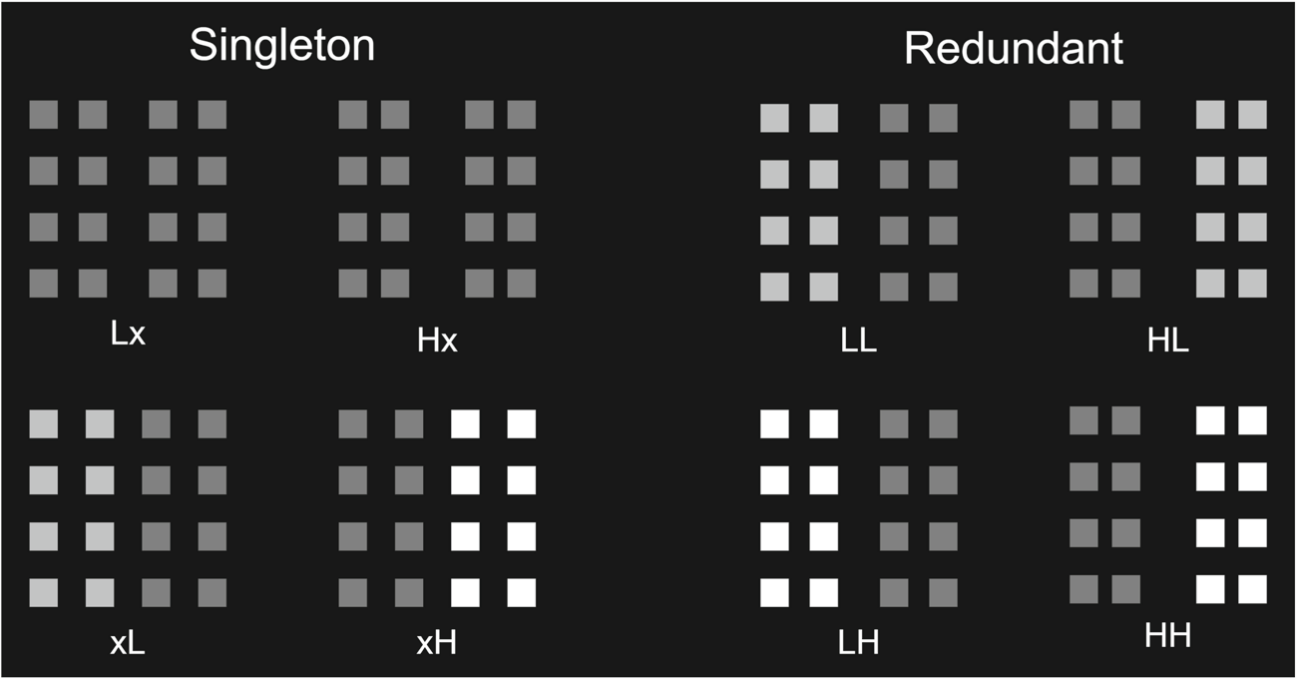
Schematic depiction of stimulus arrays from Experiment 2. On singleton trials, one of the leftmost four arrays were presented such that proximity or contrast similarity served to induce vertical or horizontal grouping orientation in isolation. On redundant trials, proximity and contrast similarity induced grouping in cooperative fashion as depicted in the rightmost four arrays. The subscripts L, H, and x denote low salience grouping, high salience grouping, and absence of grouping in channels A and B, respectively

### Method

#### Participants

Again, 60 German-speaking individuals were recruited for this online experiment. Five were excluded due to exhibiting less than 90 % overall accuracy of responding, yielding a final sample size of 55 individuals (23 females) with a mean age of 29 years (age range, 18–57 years).

#### Apparatus and stimuli

On singleton trials, the grouping orientation of the arrays was defined by either proximity *or* contrast similarity. On redundant trials, both proximity *and* contrast similarity defined the grouping orientation of the array in a cooperative fashion. In addition, two levels of grouping salience were employed for each grouping dimension: low salience proximity grouping (0.0325 height units), high salience proximity grouping (0.025 height units), low salience contrast similarity grouping (RGB value [0.5,0.5,0.5]), and high salience contrast similarity grouping (RGB value [1,1,1]). Representative stimulus arrays from Experiment 2 are depicted in Fig. 5.

#### Design

Experiment 2 embodied a double factorial two-choice RT task in which people had to identify the orientation of perceptual groups present in visual arrays as being either vertical or horizontal. For each grouping cue, two levels of salience were employed; low or high salience proximity grouping, and low or high salience contrast similarity grouping. Additionally, each trial could entail either a singleton target or two redundant targets. This yielded a total of eight stimulus conditions, defined by 2 contrast levels $\times$ 2 proximity levels $\times$ 2 redundancy levels. On redundant signal trials, the two grouping cues always indicated the same orientation (either both vertical or both horizontal). The arrays were counterbalanced for grouping orientation and the manner in which similarity grouping divided the stimulus into regions of low contrast and high contrast relative to the background. The 32 stimulus arrays (eight conditions $\times$ 4 arrays) was first cycled through in pseudo-random order once during a block of practice trials, and then three times consecutively per experimental block. A total of four practice blocks and four experimental blocks was cycled through per participant, yielding a sum total of 288 experimental trials and 128 practice trials. After each practice and experimental block, participants were encouraged but not required to take a short break. The eight experimental conditions were varied within blocks.

**Fig. 6:**
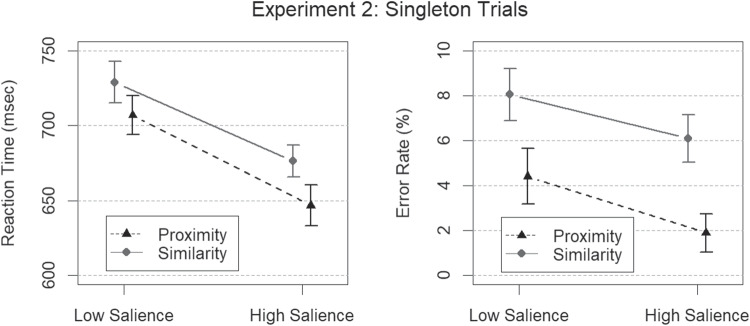
Results from Experiment 2 in terms of mean RTs (*left panel*) and mean error rates (*right panel*) from singleton trials, here depicted as a function of grouping dimension (proximity or similarity) and grouping salience (low or high salience). The *error bars* denote the 95 % HDIs of the posterior distributions of means

#### Procedure

The time-course of each experimental trial and the procedure more generally was similar to Experiment 1.

#### Data analysis

First, five participants exhibiting $< 90~\%$ overall accuracy of performance were excluded from the analysis. Outlier RTs shorter than 200 ms and longer than 1500 ms were also removed from further analysis. Then, mean-averaged RTs and error rates for singleton trials and for redundant trials were entered into separate BANOVA models. Singleton trial models incorporated the factorial predictors grouping dimension and grouping salience level. Redundant trial models incorporated the predictors proximity salience and contrast similarity salience. Incorrect responses were discarded from the analysis of mean RTs. Graphical reliability measures for mean RTs and mean error rates were again computed in terms of their 95 % HDIs.

To test the race model inequality, ten interpolated percentiles ($.05, .15, \dots , .95$) for the pertinent RT distribution CDFs and bounding sums were calculated individually for each participant following the procedures outlined in Ulrich et al. (2007). Then, the interpolated percentiles were group-averaged, and Bayesian *t* tests were computed for each of the ten group percentiles to contrast RTs on redundant signal trials with the theoretical bounding sum of the independent race model.

A SIC test and a MIC test were computed for each of 55 participants using the *sft* package (Houpt et al., 2014). The SIC test relies on a modified two-sample Kolmogorov–Smirnov test to determine if positive and negative deviations from the null hypothesis $SIC(t)=0$ are statistically reliable, as detailed in Houpt and Townsend (2010). The MIC test is based on a non-parametric adjusted rank transform test as outlined in Houpt et al. (2014). The criterion threshold of statistical significance for the SIC test and the MIC test was $\alpha = .05$. The proportion of participants exhibiting positive and negative violations of the SIC, and violations of $MIC=0$, was tested using three separate Bayesian binomial tests.

Capacity analysis was also conducted for each participant. Both positive and negative violations of the unlimited capacity assumption $C_{OR}(t) = 1$ was tested for using the Houpt–Townsend test (Houpt & Townsend, 2012). The proportion of participants exhibiting super capacity and limited capacity was tested using two separate binomial Bayesian tests. The threshold of evidential strength was set to $BF > 3$ for all Bayesian statistical tests.

### Results and discussion

Error rates for singleton trials were best captured by a model incorporating main effects of grouping dimension and grouping salience, but no factorial interaction ($BF > 5 \times 10^{24}$). Similarly, mean RT on singleton trials was also best captured by a model incorporating main effects but no interaction term ($BF > 3 \times 10^{64}$). This demonstrates that the salience manipulations for the two grouping cues were successful. Error rates and mean RTs for singleton trials are depicted in Fig. 6. Visual inspection of this figure suggests a proximity advantage, which again cannot be attributed to a trade-off between speed and accuracy.

**Fig. 7:**
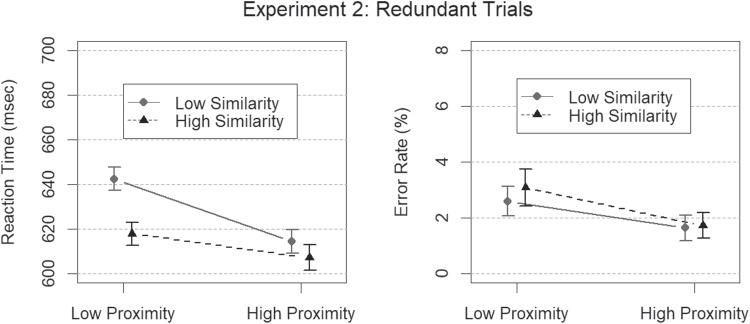
Results from Experiment 2 in terms of mean RTs (*left panel*) and mean error rates (*right panel*) for redundant trials, depicted here as a function of proximity grouping salience (high or low) and similarity grouping salience (high or low). The *error bars* represent the 95 % HDIs of the posterior distributions of means

The best model of error rates on redundant trials incorporated a main effect of proximity grouping salience, but no main effect of similar contrast grouping salience, and no proximity $\times$ contrast interaction ($BF = 87$). A main effects model gave good account of mean RTs ($BF > 10^{14}$), indicating that the two salience manipulations were effective also when grouping was redundant, but the best model of RTs on redundant trials incorporated a factorial interaction between proximity level and contrast level in addition to main effects ($BF > 3 \times 10^{14})$. Error rates and mean RTs for redundant signal trials are depicted in Fig. 7.

**Fig. 8:**
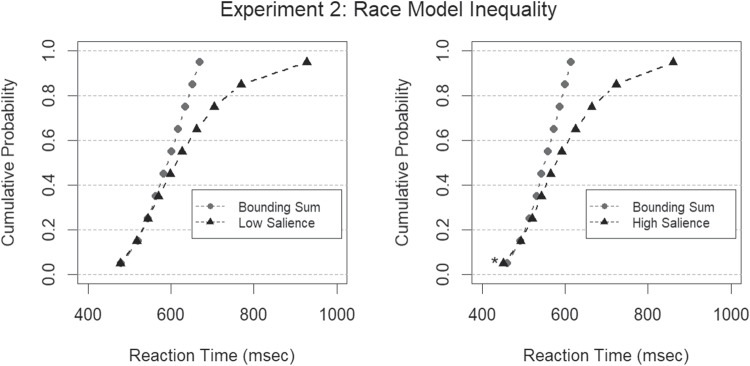
Bounding sums and empirical CDFs from redundant trials in Experiment 2 with low saliency grouping (**A**) and high saliency grouping (**B**). The *black triangles* denote the empirical CDFs from redundant trials, and the *gray circles* denote the theoretical bound of the horse race model given by the sum of the CDFs from singleton trials. The *asterisk* at the fifth percentile of high salience denotes a violated race model inequality, i.e., a Bayes factor in favor of coactive processing

The race model inequality for low salience trials was not meaningfully violated for any percentile (all $BFs < 3$). This finding is inconsistent with coactive processing of low salience proximity groups and low salience similarity groups, but for high salience trials, there was a minor inequality violation indicative of coactivation (Miller, 1982) at the smallest (.05) percentile value ($BF=4.73$). Because the race model was only violated at a single percentile, these findings are mostly inconsistent with coactive processing of proximity groups and similarity groups. Possible implications of the violation for high salience targets will be further elaborated on in the General discussion. Bounding sums and empirical CDFs are depicted in Fig. 8.

Capacity analysis based on the Houpt–Townsend test indicated limited workload capacity for redundant targets, as attested by negative violations of $C_{OR}(t)=1$ for 54 out of 55 participants with respect to low salience targets ($BF > 10^{63}$). This state of affairs also held true for high salience targets, with 52 out of 55 participants ($BF > 10^{58}$) exhibiting limited capacity. This suggests that at least one of the assumptions of the independent race model was violated. No participants exhibited super capacity for either low or high salience targets.

Eight out of 55 participants exhibited positively violated SIC for some values of $t>0$. A binomial Bayesian test showed that this was meaningfully different from 0 ($BF = 6.59$) given our threshold criterion of $\alpha = 0.05$. Only two out of 55 participants exhibited a negatively violated SIC, a finding which was not meaningfully different from 0 ($BF = 0.67$). Seven out of 55 participants further exhibited a non-zero MIC, but this finding was not significantly different from 0 ($BF = 2.87$). Six out of the eight participants who exhibited negatively violated SICs also exhibited non-zero MICs. This is to be expected since an ordering of survivor functions implies an ordering of means and vice versa. Nonetheless, for the majority of participants, both the SIC and the MIC were not different from zero.

This is in accordance with a serial processing architecture for proximity groups and contrast similarity groups with a self-terminating stopping rule for processing. Some heuristics for how to interpret the SIC and the MIC are provided in Table 1 following Houpt and Townsend (2010).

## General discussion

The goal of this research was to shed light on the time-course of proximity grouping and contrast similarity grouping in the visual pathway. To this end, Experiment 1 investigated the extent to which people can selectively attend to one of said grouping cues while ignoring the other. The following conclusions were drawn: First, judgments of proximity group orientation were in general faster and more accurate than judgments of contrast similarity group orientation (the proximity advantage). Second, the congruence effect for grouping by proximity and similar contrast levels on performance can be attributed to two types of cross-talk between grouping domains: facilitatory interaction on cooperation trials and interference on conflict trials. Finally, the congruence effect was only prominent for judgments of similarity group orientation. In other words, the flow of information in the processing chain seems to be unidirectional such that only similarity grouping is subject to congruence effects. We interpreted this finding in accordance with a serial processing account of grouping by proximity and contrast similarity (e.g., Sasaki, 2007; Trick and Enns, 1977; Quinlan and Wilton, 1998; Huang, 2015)

**Table 1:** Model predictions for serial, parallel, and coactive processing architectures

Model class	$SIC^+$	$SIC^-$	*MIC*
Serial-OR	✗	✗	✗
Serial-AND	✓	✓	✗
Parallel-OR	✓	✗	✓
Parallel-AND	✗	✓	✓
Coactive	✓	✓	✓
Experiment 2	✗	✗	✗

Serial and parallel models can incorporate self-terminating (OR) or exhaustive (AND) stopping rules for processing. The rightmost three columns list whether the model predicts a positively violated SIC ($SIC^+$), a negatively violated SIC ($SIC^-$), and a non-zero MIC. The last row in the table lists benchmarks observed for the majority of participants in Experiment 2. These results hint at a serial processing architecture for proximity groups and contrast similarity groups with a self-terminating stopping rule. Table based on Houpt and Townsend (2010)

In Experiment 2, we subjected the serial processing account of grouping by proximity and similar contrast level to an empirical test in the double-factorial paradigm. Drawing on established methodology for analyzing RTs collected in the double factorial paradigm (Townsend & Nozawa, 1995; Houpt et al., 2014; Houpt & Townsend, 2010; Wenger & Townsend, 2000) we found the following: First, the race model inequality (Miller, 1982) was not violated for low salience targets. Instead, the empirical CDF for RTs from redundant trials with low salience targets was contained within the theoretical bounding sum of the independent race model. For high salience targets, the race model inequality was on the other hand violated at the fifth percentile. Therefore, coactive processing cannot entirely be ruled out when both proximity and contrast similarity is very salient. Nonetheless, we would advise some caution when interpreting this result because of two reasons: First, a simulation study by Kiesel et al. (2007) has demonstrated that standard methods for testing the race model inequality entail a systematic bias in favor of violation, especially in the fifth to tenth percentile region. This bias stems inherently from the estimation procedures for the group CDF and bounding sum. Second, a caveat which further complicates matters is inflated Type I error rates associated with multiple comparisons. In frequentist statistics, the multiple comparisons problem can be mitigated with a suitable (e.g., Bonferroni) adjustment of alpha threshold. However, no corresponding adjustment with well-characterized statistical properties exist for the Bayesian tests employed in the present research. Kiesel et al. 2007 suggest that violations along multiple along multiple percentiles should be required to reject the race model when type I error rates are not controlled. Because only a single percentile point was violated here, we argue that our results speak against coactive processing of proximity groups and similar contrast groups, especially with respect to low salience trials. Rather, a serial or a parallel processing account seems to best capture these results from Experiment 2. However, see Townsend and Nozawa (1997) for a demonstration that inference about mental architecture based on tests of the race model inequality should not be taken at face value without aid from complementary assessments of capacity and stopping rule.

On that note, it was further observed that most participants exhibited limited workload capacity on redundant signal trials. This held true for all but one participant with respect to low salience trials, and all but three for high salience trials. This finding is inconsistent with independent parallel processing, which predicts unlimited capacity on redundant signal trials, i.e., $C_{OR}(t)=1$. Therefore, the workload capacity analysis suggests that processing of proximity groups and similar contrast groups is slower than predicted by independent racing on redundant signal trials.

Finally, analysis of means and survivor functions pertaining to RTs from redundant signal trials revealed that the majority of participants exhibited SICs and MICs which tended to zero. Following Houpt and Townsend (2010), this pattern of results is indicative of serial processing of proximity groups and contrast similarity groups with a self-terminating (OR) decision rule, as outlined in Table 1. However, the SIC was positively violated for a larger subset of participants than would be expected by chance. The following two explanations might possibly accommodate this perplexing finding from Experiment 2:

First, it might be that the analysis of SICs and MICs reveal true individual differences between participants in speeded classification of multidimensional Gestalt stimuli. For example, a subset of participants might process proximity groups and similar contrast groups in parallel, whereas others do not. Smith and Little (2018) has underscored that whenever a MIC is tested, care must be taken to ensure that the sampling distribution of the MIC is not bimodal, because the underlying parameter might be heterogeneous across participants. More careful studies of single subjects, advantageously conducted as small-N experiments using many trials and/or sessions, would be necessary to shed further light on the possibility of individual differences in orientation judgments of proximity groups and similar contrast groups, and perhaps configural visual processing tasks more broadly.

Second, it is also possible that the subset of participants with atypical performance metrics reflects the type I error rates of the SIC test and the MIC test. It has been shown by Houpt and Townsend (2010) that the model confusion probability for a serial-OR system on the basis of the SIC test and the MIC test is about 10 % when the alpha level of the Houpt–Townsend test is .05. Most usually, this results in that a serial-OR model is miscategorized as either a parallel-AND or a parallel-OR model with about equal probability, but almost never as a serial-AND model. This explanation gives a fairly parsimonious account of the findings from Experiment 2 because it only posits that certain deviations from the predictions of the serial-OR model are inevitable using the present analysis procedures and alpha threshold. However, the fairly large proportion of participants exhibiting both positive SICs and MICs, typically indicative of parallel processing with an OR-decision rule, was admittedly higher ($10.9~\%$) than would be expected by chance ($5~\%$).

In summary, Experiment 1 suggests that interactions between proximity grouping and similar contrast grouping reflects both interference and facilitation with respect to baseline. Interactions appear unidirectional in the sense that orientation judgments of similar contrast groups are affected, whereas orientation judgments of proximity groups are not. We interpreted this finding as indicative of a serial processing chain for the two grouping cues when attention is focalized towards either cue in isolation. The results of Experiment 2 provide further support the serial processing of proximity groups and similar contrast groups when attention is divided between them. Theoretically driven analyses of RTs suggest that the stopping rule for processing is self-terminating, i.e., the response mechanism is OR-gated in this task. In our view, the outcome of this research suggests that proximity groups and contrast similarity groups are processed sequentially. A subset of participants exhibited RT benchmarks more in line with parallel processing. Further research relying on more detailed assessments of individual participants are warranted to determine whether deviations from the predictions of the serial-OR model reflects true population variability in processing architecture for multidimensional Gestalt stimuli.

## Data Availability

We thank Gábor Lengyel and an anonymous reviewer for their constructive feedback on an earlier draft of this article
